# PPARγ regulates osteoarthritis chondrocytes apoptosis through caspase-3 dependent mitochondrial pathway

**DOI:** 10.1038/s41598-024-62116-w

**Published:** 2024-05-16

**Authors:** Hang Yuan, Ning Yi, Dong Li, Chao Xu, Guang-Rong Yin, Chao Zhuang, Yu-Ji Wang, Su Ni

**Affiliations:** 1 Graduate School of Bengbu Medical College, Bengbu, China; 2 Department of Orthopedics, The Affiliated Changzhou Second People’s Hospital of Nanjing Medical University, Changzhou, China; 3 Laboratory of Clinical Orthopedics, The Affiliated Changzhou Second People’s Hospital of Nanjing Medical University, Changzhou, China; 4 Graduate School of Dalian Medical University, Dalian, China; 5 Bone Disease Research and Clinical Rehabilitation Center, Changzhou Medical Center, The Affiliated Changzhou Second People’s Hospital of Nanjing Medical University, Changzhou, China

**Keywords:** PPARγ, Osteoarthritis, Chondrocytes, Apoptosis, Oxidative stress, Biochemistry, Cell biology, Diseases, Medical research, Rheumatology

## Abstract

Osteoarthritis (OA) is the most prevalent form of arthritis, characterized by a complex pathogenesis. One of the key factors contributing to its development is the apoptosis of chondrocytes triggered by oxidative stress. Involvement of peroxisome proliferator-activated receptor gamma (PPARγ) has been reported in the regulation of oxidative stress. However, there remains unclear mechanisms that through which PPARγ influences the pathogenesis of OA. The present study aims to delve into the role of PPARγ in chondrocytes apoptosis induced by oxidative stress in the context of OA. Primary human chondrocytes, both relatively normal and OA, were isolated and cultured for the following study. Various assessments were performed, including measurements of cell proliferation, viability and cytotoxicity. Additionally, we examined cell apoptosis, levels of reactive oxygen species (ROS), nitric oxide (NO), mitochondrial membrane potential (MMP) and cytochrome C release. We also evaluated the expression of related genes and proteins, such as collagen type II (Col2a1), aggrecan, inducible nitric oxide synthase (iNOS), caspase-9, caspase-3 and PPARγ. Compared with relatively normal cartilage, the expression of PPARγ in OA cartilage was down-regulated. The proliferation of OA chondrocytes decreased, accompanied by an increase in the apoptosis rate. Down-regulation of PPARγ expression in OA chondrocytes coincided with an up-regulation of iNOS expression, leading to increased secretion of NO, endogenous ROS production, and decrease of MMP levels. Furthermore, we observed the release of cytochrome C, elevated caspase-9 and caspase-3 activities, and reduction of the components of extracellular matrix (ECM) Col2a1 and aggrecan. Accordingly, utilization of GW1929 (PPARγ Agonists) or Z-DEVD-FMK (caspase-3 inhibitor) can protect chondrocytes from mitochondrial-related apoptosis and alleviate the progression of OA. During the progression of OA, excessive oxidative stress in chondrocytes leads to apoptosis and ECM degradation. Activation of PPARγ can postpone OA by down-regulating caspase-3-dependent mitochondrial apoptosis pathway.

## Introduction

Osteoarthritis (OA), also referred to as degenerative arthritis, is the most common type of arthritis globally. OA is characterized by erosion of articular cartilage, resulting in narrowing of articular space, subchondral sclerosis, subchondral cysts, synovitis and the formation of marginal osteophyte^1,2^. Chondrocyte, the sole cell type in cartilage, play an important role in cartilage repair. Numerous studies have indicated that a key event in OA is the apoptosis of chondrocytes and subsequent cartilage degeneration^3–5^. Apoptosis, influenced by various factors including oxidative stress, holds significance in tissue homeostasis. Substantial evidences have emerged that oxidative stress plays an important role in regulating chondrocytes apoptosis during the development of OA. Consequently, restoring the balance of oxidative stress is a promising way of OA treatment^6,7^.

Oxidative stress arises from reactive oxygen species (ROS), which is derived from oxygen and participate in normal intracellular signal transduction and cell degeneration. At low levels, ROS serves as a second messenger in signal transduction and homeostasis. However, when produced excessively, ROS can hamper all cellular biomolecules, including proteins, lipids and nucleic acids^8^. Mitochondria serve as the primary organelles involved in ROS production. ROS-mediated oxidative stress subsequently activates the intrinsic apoptotic pathway by releasing an array of death-promoting factors, including the mobilization of cytochrome C and caspase-9 from mitochondria to cytoplasm. The activation of caspase-9, in turn, triggers effect or caspase, such as caspase-3, leading to degradation of various substrate proteins^9^. ROS induce oxidative damage and apoptosis by disrupting the structure and function of mitochondria, thereby altering the cellular redox status.

Apoptosis, a form of programmed cell death, is essential for the normal development of the body and the maintenance of homeostasis. It is associated with the exogenous pathway, mediated by cell death receptor, and the endogenous pathway, mediated by mitochondria^10^. The mitochondrial-mediated apoptotic pathway initiates with the loss of mitochondrial membrane potential, followed by the release of cytochrome C and the cleavage of caspase-3, eventually culminating the formation of apoptotic bodies^11^.

Peroxisome proliferator-activated receptor gamma (PPARγ) is a ligand-induced transcription factor known to involve in normal cell function. Upon activation, PPARγ forms a heterodimer with the retinol X receptor, binding to specific reaction elements and promoting the expression of target genes^12^. PPARγ belongs to the nuclear hormone receptor super family and is believed to regulate many signaling pathways under diverse pathological conditions. Activation of PPARγ is thought to have protective effects against cell apoptosis. After activation by specific ligands, PPARγ translocates to other activated PPAR response elements to initiate target gene transcription. Previous study has shown that PPARγ is a key regulator of cartilage health, and the lack of PPARγ accelerations the onset of spontaneous OA^13^. Given the crucial etiological role of apoptosis in OA, this study aims to explore the regulatory mechanism of PPARγ in the apoptosis of OA chondrocytes.

## Materials and methods

### Cell harvest and culture

Methods for harvesting chondrocytes was conducted as described previously^14^. In brief, human cartilage specimens were obtained from 50 patients who were diagnosed with knee OA (of Kellgren–Lawrence (KL) score 3–4) according to the 1985 criteria of the American Rheumatism Association by total knee replacement. The relatively normal cartilage and damaged cartilage specimens were gathered for tissue analysis and digested for in vitro investigations. Specimens were sliced into small pieces, washed with phosphate-buffered saline (PBS) (Tianjin Haoyang Biotech. Co., Ltd, Tianjin, China) for three times, and digested with 2 mg/ml collagenaseII (Sigma-Aldrich, St Louis, MO, USA) at 37 °C overnight. The digested cartilage was collected and centrifuged at 1000 rpm for 10 min. The pellet was re-suspended, filtered through a 70 μm cell strainer, and finally cultured in Dulbecco’s modified Eagle’s medium (DMEM) with 100 U penicillin and 100 μg/streptomycin (Nanjing KeyGEN Biotech. Co., Ltd, Nanjing, China) supplemented with 10% fetal bovine serum (FBS) (Tianjin Haoyang Biotech. Co., Ltd, Tianjin, China) in a standard cell culture chamber containing 5% CO_2_.Confluent chondrocytes were split in 1:3 ratio up to passage 1 and these cells were used for subsequent experiments. This study was reviewed and approved by the ethics committee of the No.2 People’s Hospital of Changzhou, Jiangsu, China ([2020] KY221-01).Written informed consent was obtained from all participants included in the study. All the methods used in this study were carried out in accordance with the approved protocol and guidelines.

### Cell treatment

Chondrocytes were cultured in various formats: 96-well plates for cell proliferation assay, 6-well plates for cell viability and cytotoxicity, cell apoptosis detection, measurement of ROS, detection of nitric oxide (NO), mitochondrial membrane potential (MMP) measurement, cytochrome C release and protein experiments, and 6-cm dishes for mRNA extraction. Upon reaching confluence, OA chondrocytes were pretreated with 10 μM GW1929 (Med Chem Express, Shanghai, China) or 10 μM Z-DEVD-FMK (AdooQ Bioscience, Irvine, CA, USA) for 1 h.

### Cell proliferation

Cell proliferation was determined by 3-(4,5-dimethylthiazol-2-yl)-2, 5-diphenyltetra-zolium bromide reduction (MTT) assay (Beyotime Biotechnology, Shanghai, China)^4^. In brief, the indicated cells were seeded in 96-well plate at a density of 2 × 10^4^ per well. Before the end of the experiment, 20 μL MTT was added and the plate was incubated at 37 °C for 4 h. Subsequently, 150 mL DMSO was added to dissolve formazan and the absorbance was measured at 570 nm by the microplate reader (Elx808 Bio-Tek Instruments, Winooski, VT, USA).

### Cell staining

Chondrocytes were fixed in 4% paraformaldehyde for 30 min and stained with crystal violet (Beyotime Biotechnology, Shanghai, China) for 3 h, followed by slowly washing with running water. Formed colonies were observed under an inverted microscope (GX41, OLYMPUS, Tokyo, Japan)^15^.

### Cell viability and cytotoxicity

Chondrocytes were washed once with PBS and then exposed to calcein AM/PI test working solution. The plate was incubated in the dark at 37 °C for 30 min, after which the calcein AM green (excitation 494 nm/emission 517 nm) and PI red (excitation 535 nm/emission 617 nm) fluorescence was observed using a fluorescence microscope (CX41-32RFL, OLYMPUS, Tokyo, Japan)^15^.

### Cell apoptosis

To quantify the percentage of chondrocytes undergoing apoptosis, the FITC Annexin Vapoptosis detection kit (BD Pharmingen, San Diego, CA, USA) was used as described previously^16^. Briefly, chondrocytes were harvested, washed twice with cold PBS, and then re-suspended in 100 μL binding buffer into which 5 μL of FITC Annexin V and 5 μL of propidium iodide (PI) were added. After incubating at room temperature for 15 min avoiding light, 400 μL binding buffer was added, and the chondrocytes were analyzed with a FACScan flow cytometer (BD Biosciences, San Jose, CA, USA).

### Measurement of ROS

The intracellular ROS level was assessed using a chemical fluorescence method with 2,7-dichlorofuorescin diac-etate (DCFH-DA, Beyotime Biotechnology, Shanghai, China)^15^. Briefly, the cell culture medium was discarded, and the chondrocytes were washed with DMEM. Subsequently chondrocytes were incubated with DCFH-DA for 30 min at 37 °C in the dark. After washing with PBS, chondrocytes were observed using a fluorescence microscope (CX41-32RFL, OLYMPUS, Tokyo, Japan).

### Detection of NO

The NO production in chondrocyte culture supernatants was measured using the Griess method (Promega, Milan, Italy)^14^. At the end of stimulations, NO production in the sample’s supernatants was examined by adding an equal volume of Griess reagent according to the manufacturer’s instruction. The absorbance at 570 nm was measured by the microplate reader (Elx808 Bio-Tek Instruments, Winooski, VT, USA).

### Determination of cytochrome C

Cytochrome C release was determined by an ELISA kit (Thermo Fisher Scientific, USA) according to the manufacturer’s instructions. In brief, chondrocytes were washed and re-suspended in cell lysis buffer at a concentration of 1.5 × 10^6^ cells/mL. Cells were lysated for 1 h at room temperature with gentle mixing, and then centrifuged at 1000 g for 15 min. The supernatant was used for detection. Next, 50 μL of calibrator diluents, standard, control and sample were added to each well of a 96-well plate. The plate was incubated for 0.5 h at 37 °C, then washed and dried. After that, 50 μL of conjugate was added to each well to incubate again for 0.5 h at 37 °C. Following the rinse of washing buffer, a100 μL of substrate solution was added to each well and the plate was incubated for 10 min at 37 °C in dark. After adding 50 μL of stop solution, the optical density was determined within 30 min, and absorbance at 450 nm was measured using a microplate reader (Elx808 Bio-Tek Instruments, Winooski, VT, USA).

### MMP measurement

The medium of the chondrocytes was aspirated, and the cells were incubated with 5,51,6,61-tetrachloro-1,11,3,31 tetraethylbenzimidazolyl carbocyanine iodide (JC-1) at a 1× diluted in assay buffer for 15 min at 37 °C in an incubator according to the manufacturer’s instruction (Thermo Fisher Scientific, USA)^15^. The dyes were dissolved in dimethyl sulfoxide (DMSO), with the percentage of the organic solvent in the samples never exceeding1%vol/vol. After the incubation, the cells were washed twice with 1× assay buffer, and the suspensions were transferred in triplicates to a black 96-well plate. The red (excitation 550 nm/emission 600 nm) and green (excitation 485 nm/emission 535 nm) fluorescence was observed with a fluorescence microscope (CX41-32RFL, OLYMPUS, Tokyo, Japan).

### Quantitative real-time reverse transcription polymerase chain reaction (qRT-PCR)

Total RNA was extracted from samples using TRIzol (Invitrogen, Carlsbad, CA, USA). High capacity cDNA reverse transcription kit (Applied Biosystems, Foster City, CA, USA) was used to reverse transcribe total RNA (1 μg) as described previously^17^. Aggrecan, collagen type II (Col2a1), inducible nitric oxide synthase (iNOS), PPARγ,caspase-9, caspase-3and glyceraldehyde 3-phosphate dehydrogenase (GAPDH) were amplified using SYBR select master mix (Applied Biosystems, Austin, TX, USA) in a Bio-Rad iQ5.The specific primer sequences are presented in Table 1. The data were calculated by comparative threshold cycle method.

**Table 1:** Primer sequences for qRT-PCR.

Gene	Forward (5' → 3')	Reverse (5' → 3')
Aggrecan	GACTTCCGCTGGTCAGATGG	CGTTTGTAGGTGGTGGCTGTG
Col2a1	GGGATCGTGGTGACAAAGGT	CTGGGCAGCAAAGTTTCCAC
iNOS	CGTGGAGACGGGAAAGAAGT	GACCCCAGGCAAGATTTGGA
PPARγ	TGCATTCTGCTTAATTCCCTTTCC	GTGTCAACCATGGTCATTTCGTTA
Caspase-9	ATTGGTTCTGGAGGATTTGGTGATG	ATGCTCAGGATGTAAGCCAAATCT
Caspase-3	GGCGGTTGTAGAAGAGTTTCG	TCACGGCCTGGGATTTCAAG
GAPDH	GAAAGCCTGCCGGTGACTAA	GCCCAATACGACCAAATCAGAGA

### Western blotting

Human cartilage tissues (after liquid nitrogen grinding) or chondrocytes were harvested and lysed in RIPA buffer (BostonBioproducts, MA, USA) for total protein extraction as we described before^16^. Equal amounts of protein (10 μg) were boiled and subjected to electrophoresis on 10% sodium dodecyl sulfate–polyacrylamide gels and transferred to a polyvinylidene fluoride (PVDF) membrane (Millipore, MA, USA). We used to cut the target band before hybridization based on the expected molecular size. Following a 1-h block in trisbuffered saline with Tween-20 containing 5% nonfat milk, the PVDF membrane was probed with primary antibodies (Cell Signaling Technology, Danvers, MA, USA; diluted in 1:1000) overnight at 4 °C, and incubated with horseradish peroxidase (HRP) conjugated secondary antibody (Cell Signaling Technology, Danvers, MA, USA; diluted in 1:5000) for 1 h at room temperature. Blots were detected with the enhanced chemiluminescence (ECL) assay kit (Santa Cruz Biotechnology, USA). The β-Actin signal was used as an internal loading control, and relative expression levels were quantified using Quantity One (Bio-Rad Laboratories, Hercules, CA, USA).

### Chromatin immunoprecipitation (ChIP)

Cultured human normal chondrocytes (iCell Bioscience, Inc. Shanghai, China) were subjected to 10 ng/ml IL-1β or pretreated with10 μM GW1929 then 10 ng/ml IL-1β or vehicle (PBS solution) for 12 h. A total of 10^7^ cells were fixed and cross-linked in fresh 1% formaldehyde for 10 min and then quenched with 2.5 M glycine for 5 min at room temperature. Cells were then harvested and suspended in lysis buffer. Simple ChIP Enzymatic Chromatin IP Kit (Cell Signaling Technology, Danvers, MA, USA) was used according to the manufacturer’s instructions. Chromatin was digested with micrococcal nuclease, sheared by sonication and then lysates were clarified by centrifugation at 10,000 rpm for 10 min at 4 °C. The supernatant was incubated with ChIP-grade rabbit antihuman PPARγ polyclonal antibody (Cell Signaling Technology, Danvers, MA, USA) or normal immunoglobulin G (IgG) overnight at 4 °C with rotation. After being pulled down with protein G agarose beads, the target proteinDNA complexes were sequentially washed several times and reverse cross-linked to elute DNA for the subsequent experiment. DNA was purified and real-time PCR was performed to evaluate the ChIP-enriched DNA. Primer sequences for detecting PPARγ-binding iNOS promoter region were 5′- TTTATGACTGTGACTGCCAGGG -3′ (forward) and 5′- TGAACTGCCACCTTGGACTT -3′ (reverse).

### Statistical analysis

Data shown in our study were represented as means ± SD from a minimum of three independent experiments. One-way ANOVA, followed by Bonferroni test, was conducted for comparisons between two groups. Significantly difference was considered when *P* < 0.05.

## Results

### Decreased activity of PPARγ in OA cartilage

**Figure 1:**
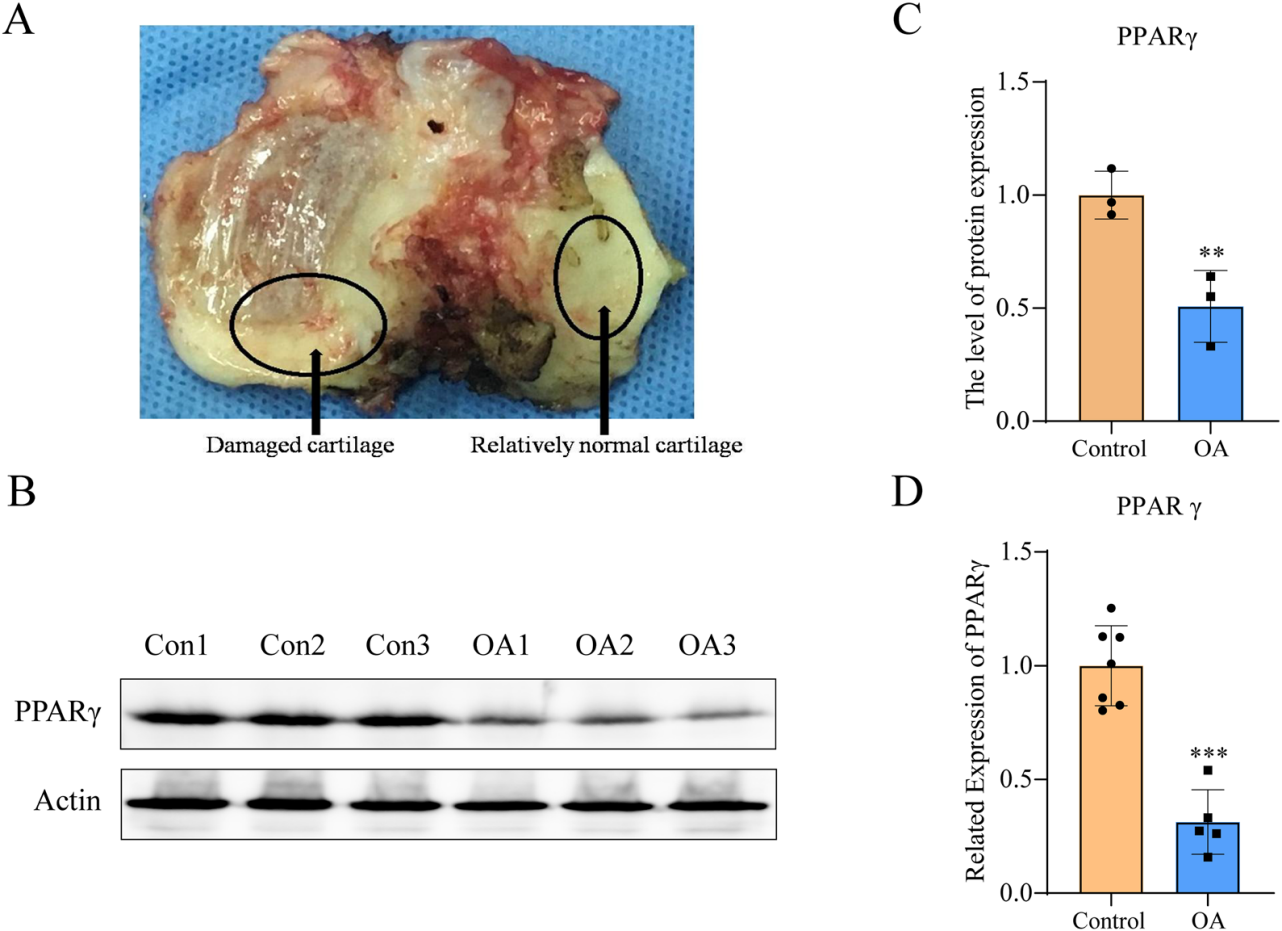
Down-regulation of PPARγ in OA cartilage. (**A**) Representative image of cartilage from patient with total knee replacement showing relatively normal cartilage (Con) and damaged cartilage (OA); protein expression (**B**) and quantification data of PPARγ (**C**); (**D**) mRNA expression of PPARγ. **P < 0.01, ***P < 0.001 versus Con.

To explore the changing of PPARγ in degenerative cartilage of OA, cartilage specimens were categorized into relatively normal cartilage (Con) and damaged cartilage (OA) (Fig. 1A) from patients undergoing total knee replacement. Western blotting (Fig. 1B,C) and qRT-PCR (Fig. 1D) analysis revealed that PPARγ was down-regulated in damaged cartilage.

### Cell proliferation and viability of OA chondrocytes

**Figure 2:**
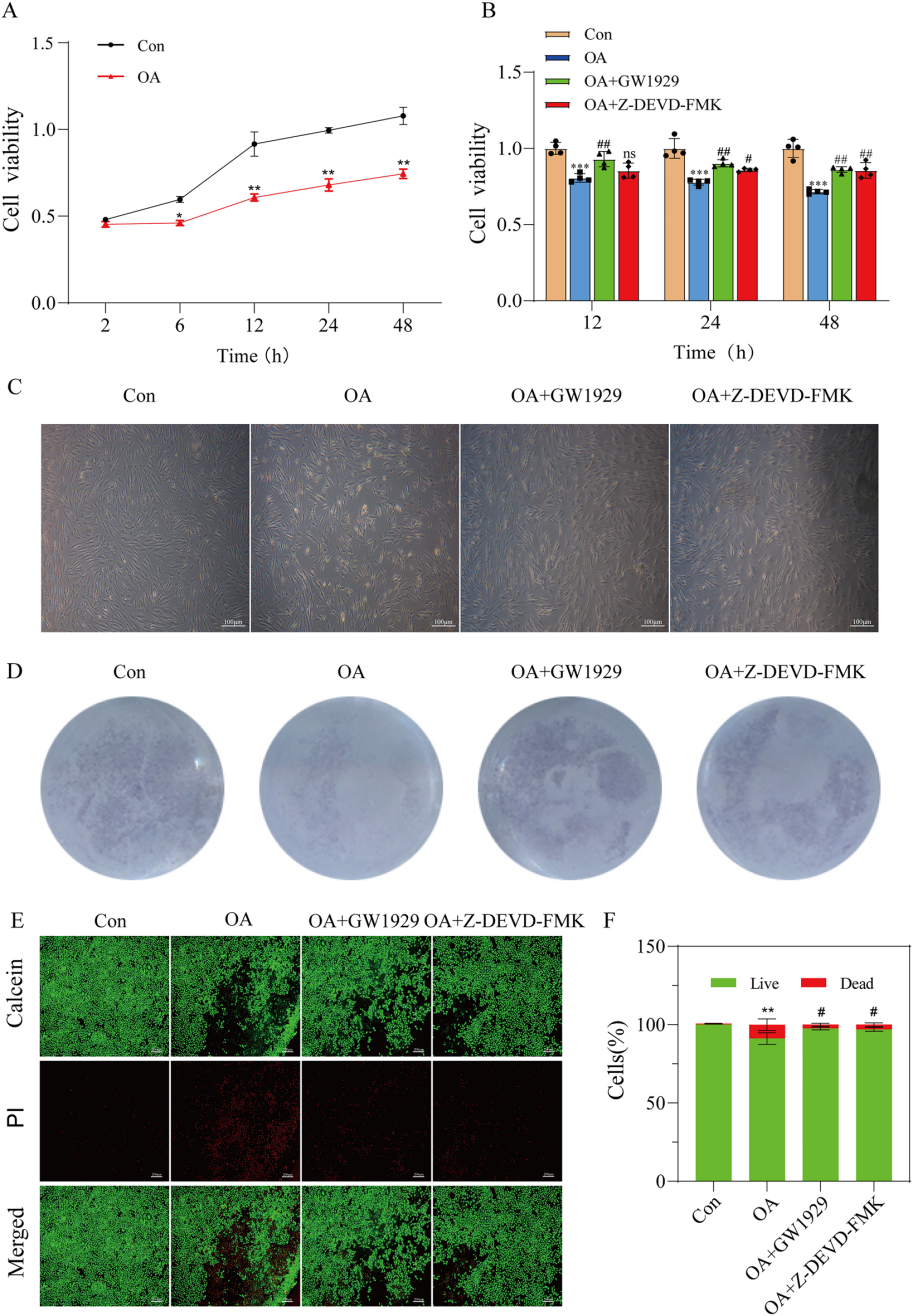
Cell proliferation and viability of OA chondrocytes. (**A**) Control chondrocytes and OA chondrocytes were cultured in vitro for 2 h, 6 h, 12 h, 24 h and 48 h and cell proliferation was detected by MTT. (**B**) OA chondrocytes were induced by different treatments and cell proliferation was detected by MTT. (**C**) Observation of cell state. (**D**) Crystal violet staining. (**E**) Control chondrocytes and OA chondrocytes were cultured in vitro for 24 h and dyed by Calcein AM/PI. (**F**) Quantification of fluorescence intensity. Results are presented as means ± standard deviation of three independent experiments. *P < 0.05, **P < 0.01, ***P < 0.001 versus Con. ^#^*P* < 0.05, ^##^*P* < 0.01 versus OA.

We cultured relatively normal cartilage (NC) and damaged cartilage (OA) in vitro, respectively, and proliferation of chondrocytes was detected at 2 h, 6 h, 12 h, 24 h, and 48 h. Compared with the NC group, the proliferation of OA chondrocytes decreased significantly (Fig. 2A). Treatment with 10 μM GW1929 or 10 μM Z-DEVD-FMK resulted in an increased proliferation of OA chondrocytes (Fig. 2B). Direct observation under an inverted microscope, crystal violet staining, and cell viability and cytotoxicity tests indicated an enhanced survival rate of pretreated chondrocytes (Fig. 2C–F). These findings suggested that activation of PPARγ could improve the viability of OA chondrocytes and might be involved in down-regulation of caspase-3 dependent apoptosis.

### Cell apoptosis of OA chondrocytes

**Figure 3:**
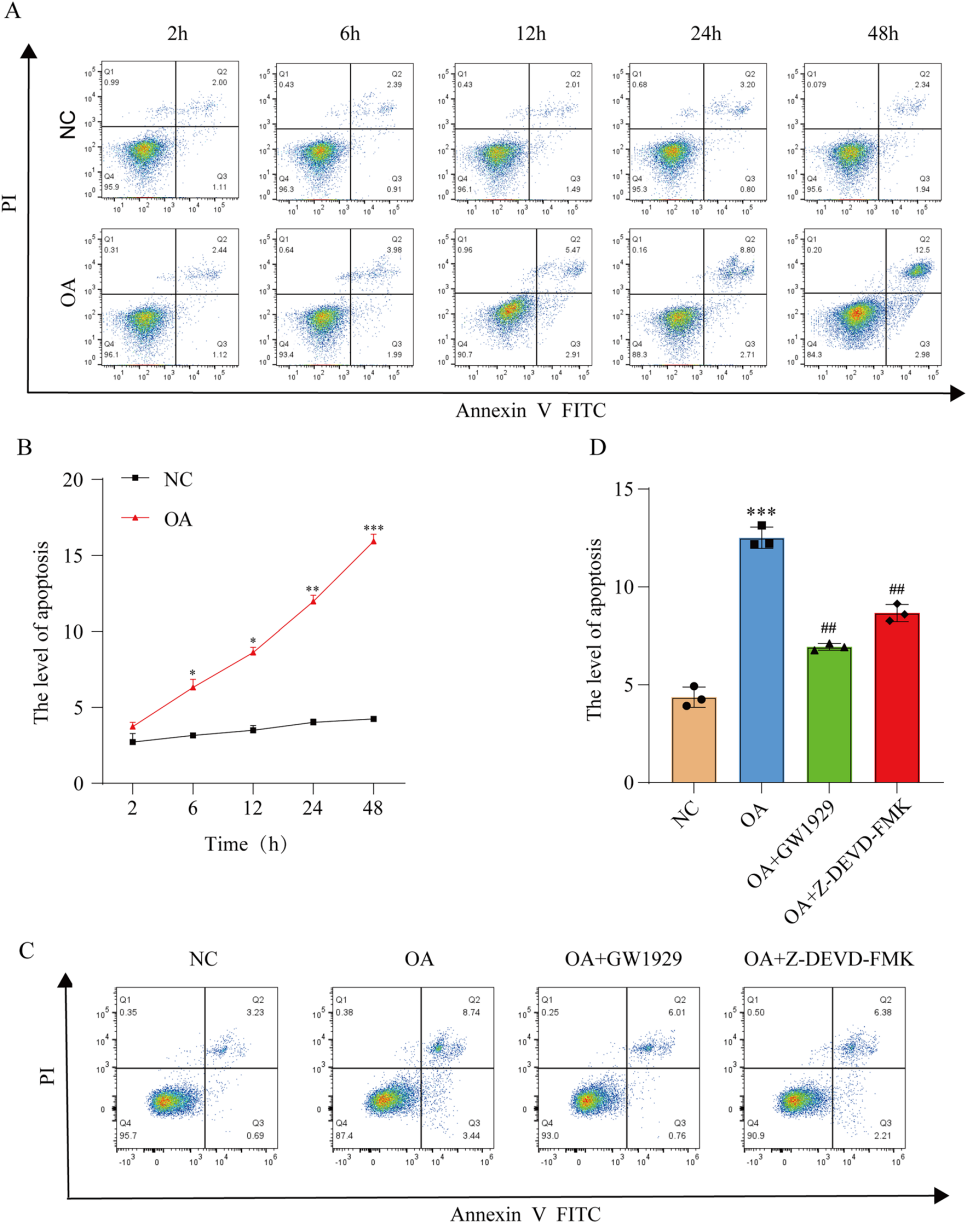
Cell apoptosis of OA chondrocytes. (**A**) Control chondrocytes and OA chondrocytes were cultured in vitro for 2 h, 6 h, 12 h, 24 h and 48 h. (**C**) OA chondrocytes were induced by different treatments. FITC annexin V/PI staining and flow cytometry assays were used to detect cell apoptosis. Control chondrocytes cultured in DMEM were used as control. (**B**) and (**D**) Quantification of apoptosis in different groups. Results are presented as means ± standard deviation of three independent experiments. *P < 0.05, **P < 0.01, ***P < 0.001 versus Con. ^##^*P* < 0.01 versus OA.

Chondrocyte apoptosis was further investigated using flow cytometry. Normal chondrocytes and OA chondrocytes cultured in vitro were examined at different time points (2 h, 6 h, 12 h, 24 h, and 48 h) (Fig. 3A), Normal chondrocytes cultured in DMEM were used as control. The percentage of apoptotic cells in quadrants Q2 and Q3 was calculated. A substantial increase in the apoptosis rate of OA chondrocytes was observed, consistent with the findings from the cell proliferation assay (Fig. 3B).When treated with 10 μM GW1929 or 10 μM Z-DEVD-FMK, OA chondrocytes apoptosis was relieved (Fig. 3C,D). These results further strengthen the suggestion that enhancing PPARγ activity could inhibit chondrocytes apoptosis, which is somehow related to caspase-3.

### Oxidative stress changed mitochondrial function of OA chondrocytes

**Figure 4:**
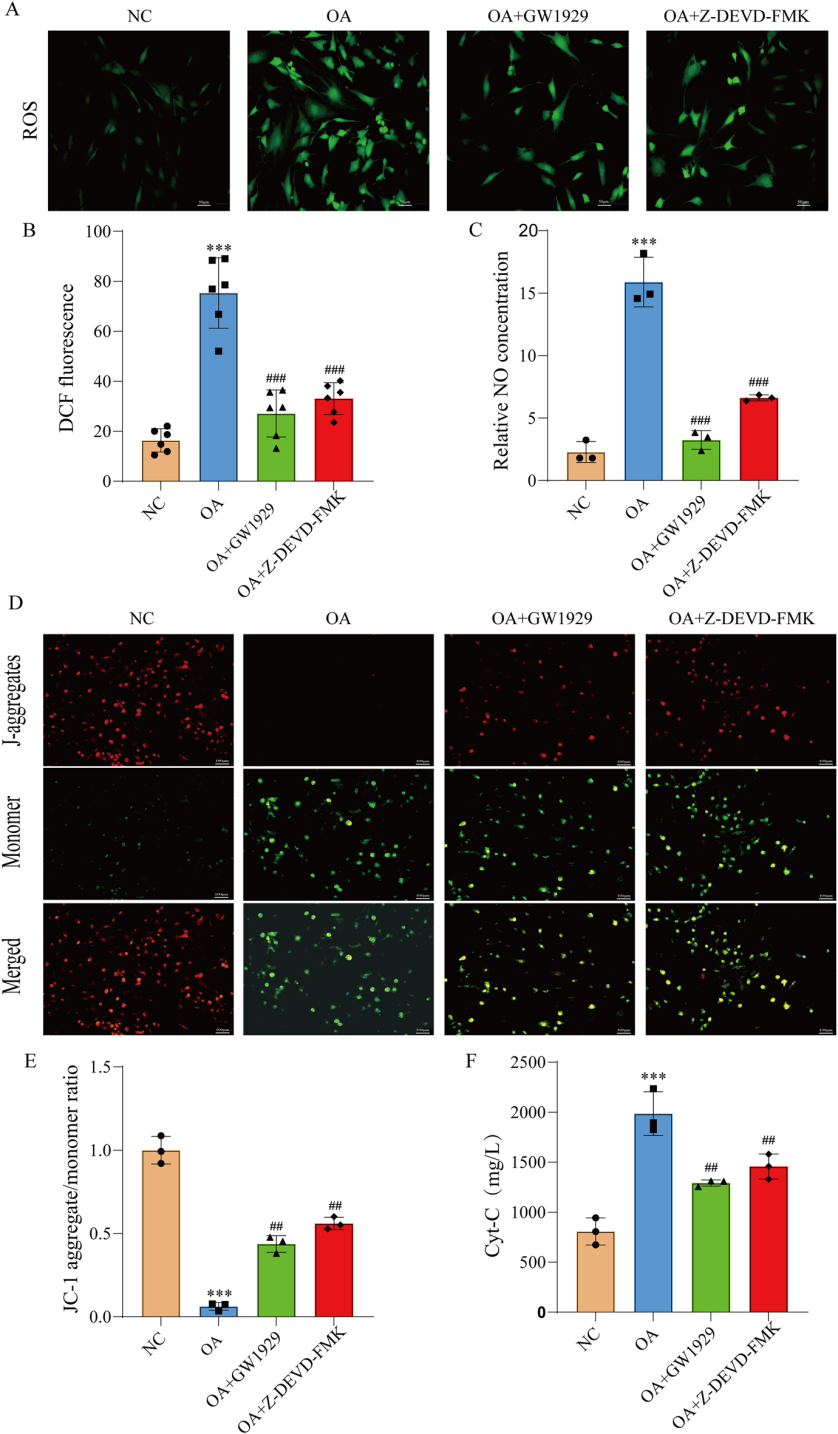
Detection of mitochondrial function. (**A**) Intracellular ROS; (**B**) fluorescence intensity; (**C**) NO; (**D**) MMP; (**E**) fluorescence intensity; (**F**) cytochrome C. Control chondrocytes cultured in DMEM were used as control. Results are presented as means ± standard deviation of three independent experiments. ***P < 0.001 versus Con. ^##^*P* < 0.01, ^###^*P* < 0.001 versus OA.

To evaluate the difference of oxidative stress among different chondrocytes, we detected the intracellular ROS and NO production. The level of intracellular ROS in OA chondrocytes was significantly higher than that in the NC group (Fig. 4A). Additionally, the content of NO secreted by OA chondrocytes was also significantly increased compared to the NC group (Fig. 4B), indicating the presence of excessive oxidative stress in OA chondrocytes. Mitochondria are not only the main source of ROS in chondrocytes but also the primary target of ROS. Impairment of mitochondrial function leads to a metabolic disorder in chondrocytes, which in turn result in excessive apoptosis. Evaluation of MMP and cytochrome C levels revealed a significant reduction in MMP in OA chondrocytes compared to the NC group (Fig. 4E), indicating the increased mitochondrial permeability and excessive release of cytochrome C (Fig. 4D). These signals initiated the mitochondrial apoptosis pathway. When treated with 10 μM GW1929 or 10 μM Z-DEVD-FMK for 1 h, oxidative stress in OA chondrocytes decreased and the functional integrity of mitochondria was protected (Fig. 4A–E).These results suggested that a negative correlation between PPARγ activity and oxidative stress. Maintaining PPARγ activity in chondrocytes was crucial to mitochondrial function, and indirectly affected caspase-3 activity.

### PPARγ regulates OA chondrocytes apoptosis through caspase-3 dependent pathway

**Figure 5:**
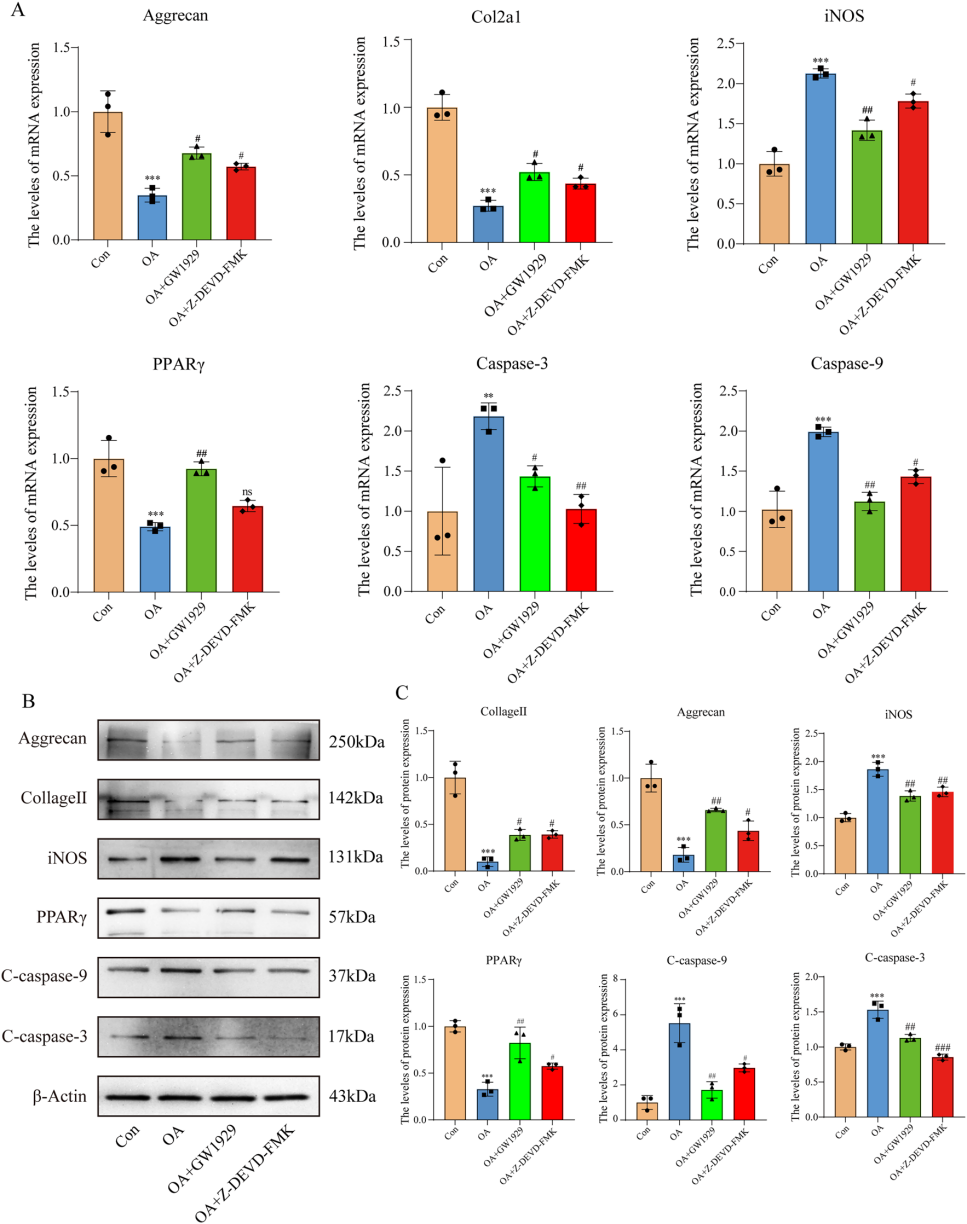
Expressions of relative genes and proteins. (**A**) Control chondrocytes and treated OA chondrocytes were harvested and total RNA was extracted, followed by qRT-PCR for detection of aggrecan, Col2a1, iNOS, PPARγ, caspase-9 and caspase-3gene expression levels. (**B**) Control chondrocytes and treated OA chondrocytes were harvested and total protein was extracted, western blotting was used to detect the expression of aggrecan, Collage II, iNOS, PPARγ, caspase-9 and caspase-3. (**C**) Quantification data of western blotting. The relative protein levels were normalized to the level of the internal control, β-Actin and presented as fold changes relative to the control group (the level of the control group was set as 1). Results are presented as means ± standard deviation of three independent experiments. **P < 0.01, ***P < 0.001 versus Con. ^#^*P* < 0.05, ^##^*P* < 0.01, ^###^*P* < 0.001 versus OA.

In order to clarify the relationship between PPARγ and caspase-3 in OA chondrocytes, we conducted qRT-PCR and western blotting analyses. As we assumed, compared with NC group, PPARγ activity in OA chondrocytes was inhibited, iNOS gene and protein levels were highly expressed, along with the increased mRNA and protein levels of caspase-9 and caspase-3, where as the expression of ECM components Col2a1 and aggrecan significantly decreased. Upon treatment with GW1929 to enhance PPARγ activity, iNOS gene and protein expressions were down-regulated. Meanwhile, the expressions of caspase-9 and caspase-3 decreased, and Col2a1 and aggrecan expressions were up-regulated. After Z-DEVD-FMK treatment, compared with OA group, the gene expression of PPARγ showed no significant change, but its proteolysis level was up-regulated, suggesting inhibition of apoptosis could indirectly enhance PPARγ activity (Fig. 5A–C). These results suggested that PPARγ might regulate chondrocytes apoptosis and affect chondrocytes metabolism by regulating oxidative stress through caspase-3 dependent pathway.

## Discussion

OA is one of the most common chronic diseases characterized by the degradation of extracellular matrix (ECM) of cartilage^18^. ECM is mainly composed of proteoglycan and collagen II, which plays an important role in preserving the internal stability and integrity of cartilage. The initial stage of cartilage degeneration involves the loss of proteoglycan, followed by the catabolism of collagen II fibers, which eventually leads to the partial or complete loss of ECM^19,20^. Recent studies have revealed that OA chondrocytes exhibit loss of mitochondrial function, which is a typical marker of apoptosis^21,22^. This investigation confirmed that, in comparison to human normal chondrocytes, OA chondrocytes exhibit lower PPARγ activity and a higher apoptotic rate, culminating in ECM loss. Apoptosis was believed to tightly involved in the occurrence and development of OA. Our previous study has proved oxidative stress could affect the apoptosis of chondrocytes which is related to caspases family^23^. In the present research, we provide further elucidation on the regulatory relationship between PPARγ and caspase-3.

Mitochondria generate ROS as metabolic byproducts of the respiratory electron transport chain. Normally, a delicate equilibrium exists between ROS production and the antioxidant system. However, excessive ROS can disrupt this balance, instigate oxidative stress and ultimately lead to apoptosis^24^. At low concentration, intracellular ROS play an important role in protecting cell signaling pathways, and can even induce cell proliferation and differentiation by regulating protein expression. However, overproduced ROS could compromise MMP. That is consistent with our findings in OA chondrocytes. Conformational changes in mitochondrial membrane would open mitochondrial permeability transition pore and increase mitochondrial membrane permeability, resulting in release of cytochrome C. And these results aligned with prior previous research^25,26^.

Apoptosis, a physiological mechanism in organisms, represents a highly organized process crucial for maintaining internal environmental stability. It is characterized by the gradual activation of precise pathways leading to specific biochemical and morphological changes^27^. The initial stages of apoptosis involve alterations in redox potential, cell contraction and the loss of membrane lipid asymmetry. In OA, apoptosis initiates cartilage degradation, and is accelerated by overproduction of ROS^28^. Evidence has shown that ROS-induced mitochondria malfunction could directly activate caspase-dependent pathway and promote apoptosis^29^, which is consistent with our results. Caspases, a family of specific cysteine proteases, are critical mediators of apoptosis, which can be activated by oxidative stress. Activation of caspase-9 leads to the mitochondrial release of cytochrome C which in turn results in the activation of caspase-3. Caspase-3 is a member of the caspase signaling pathway and the most important executor of cell apoptosis, which can be activated by mitochondrial apoptotic pathways^30^.

PPARγ is a nuclear receptor protein and a transcription factor regulating gene expression. When activated by ligands, PPARγ regulates transcription by binding to a specific DNA sequence element in the promoter of the target gene^31^. PPARγ is currently considered to be an appealing therapeutic target against OA-related cartilage degradation. Existing evidence highlights that the activation of PPARγ can effectively reduce the apoptotic rate of chondrocytes, alleviate OA-related catabolism and mitigating the progression of cartilage injury in experimental OA models^32–35^. In this experiment, we used cartilage and chondrocytes of OA patients as research subjects, and the results consistently affirm that activation of PPARγ could alleviate apoptosis and protect chondrocytes.

Previous studies have revealed that PPARγ is able to suppress oxidative stress by transcriptional repression of iNOS^36–39^, which up-regulation would induce the production of NO leading to excessive oxidative stress^40^. Recent years, studies have substantiated the significant elevation of NO levels in the serum and synovial fluid of OA patients. In animal models of OA, chondrocytes apoptosis and matrix consumption are correlated with NO production^41,42^. In this study, we also observed an increase in NO secretion corresponding with elevated iNOS expression in OA chondrocytes. This excessive NO level induced an unfavorable production of mitochondrial ROS, exacerbated oxidative stress, and in turn hampered ECM synthesis and promoted chondrocytes apoptosis. NO, serving as an intracellular messenger, plays a crucial role in various pathophysiological processes^43^. ROS overload caused by NO accelerates the process of chondrocytes apoptosis by reducing MMP, prompting the opening of mitochondrial permeability transition pore and facilitating the release of cytochrome C. This process disrupts the respiratory chain, resulting in decreased ATP levels^44^. Energy metabolism disorder promotes the progress of OA by inhibiting the proliferation of chondrocytes, destroying the stability of cartilage-specific ECM, inhibiting the synthesis of collagen II and proteoglycan, culminating in cartilage injury^45^. In contrast, we employed an agonist to increase the activity of PPARγ, aiming to down-regulate the expression of iNOS, and subsequently inhibit NO secretion. This intervention resulted in a lower level of ROS in mitochondria, maintaining stable mitochondrial function. Consequently, there was a reduction in the proteolysis of caspase-9 and caspase-3, fostering a decreased chondrocytes activity and ECM synthesis.

## Conclusions

**Figure 6:**
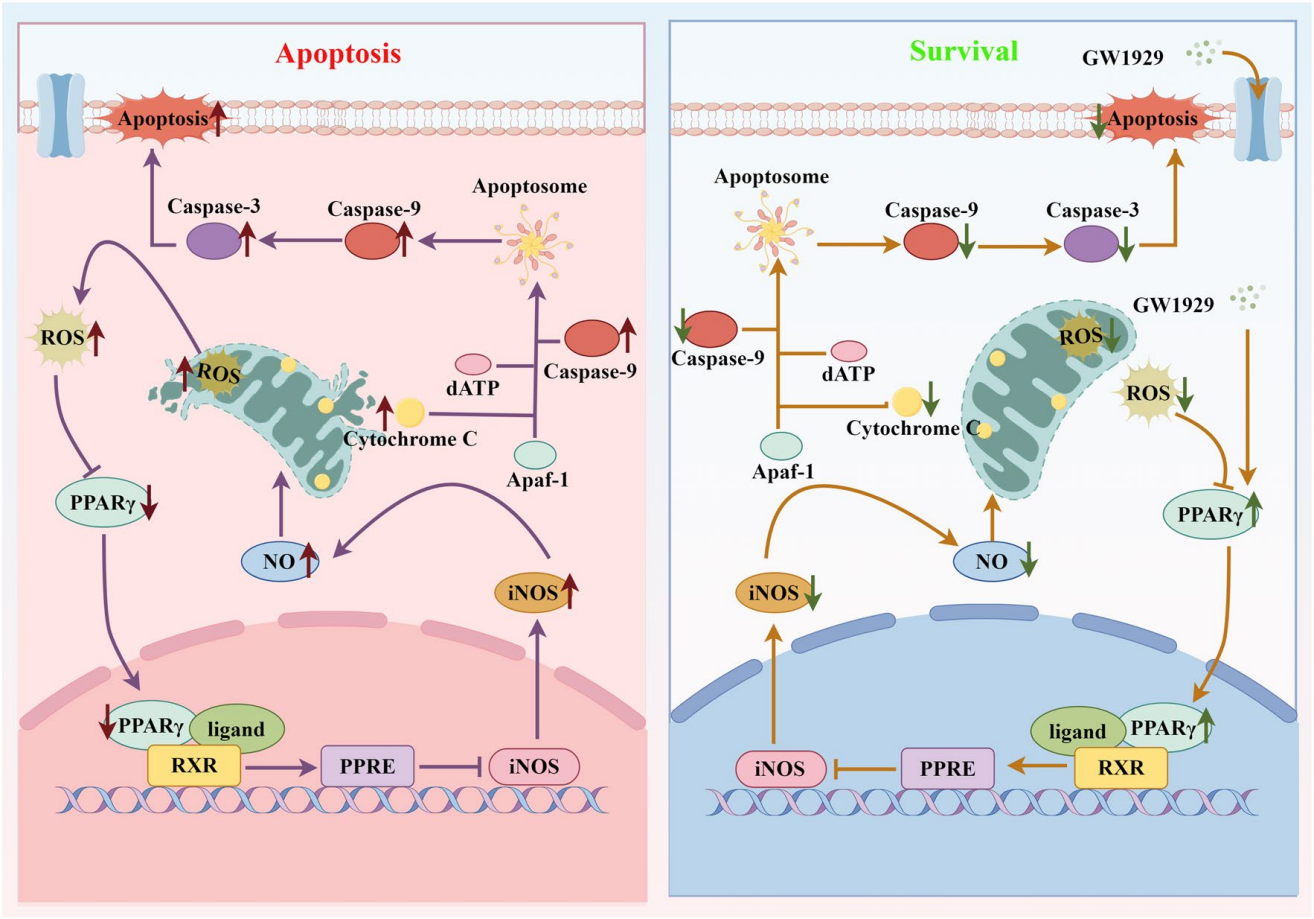
Schematic depiction of the mechanisms of PPARγ regulates OA chondrocytes apoptosis through caspase-3 dependent mitochondrial pathway (By Figdraw). PPARγ regulates oxidative stress induced OA chondrocytes apoptosis by down-regulation of iNOS and reducing NO production, which results in the inhibition of ROS production, thereby maintaining the stability of MMP and preventing cytochrome C overflow, finally blocking the activation of caspase-9 and caspase-3.

In conclusion, as shown in Fig. 6, our present study revealed that activation of PPARγ effectively reversed the apoptosis of OA chondrocytes by down regulating iNOS activity, reducing NO synthesis and production of ROS in mitochondria, maintaining mitochondrial function, and so as to lessen caspase-3 dependent mitochondrial apoptotic pathway. These findings provide a novel insight into the role of PPARγ in the pathophysiology of OA.

### Supplementary Information

Supplementary Information.

## Data Availability

The datasets used and analyses during the current study available from the corresponding author on reasonable request.
