# Author Correction: PPARγ regulates osteoarthritis chondrocytes apoptosis through caspase-3 dependent mitochondrial pathway

**DOI:** 10.1038/s41598-024-64106-4

**Published:** 2024-06-10

**Authors:** Hang Yuan, Ning Yi, Dong Li, Chao Xu, Guang‑Rong Yin, Chao Zhuang, Yu‑Ji Wang, Su Ni

**Affiliations:** 1 Graduate School of Bengbu Medical College, Bengbu, China; 2 Department of Orthopedics, The Affiliated Changzhou Second People’s Hospital of Nanjing Medical University, Changzhou, China; 3 Laboratory of Clinical Orthopedics, The Affiliated Changzhou Second People’s Hospital of Nanjing Medical University, Changzhou, China; 4 Graduate School of Dalian Medical University, Dalian, China; 5 Bone Disease Research and Clinical Rehabilitation Center, Changzhou Medical Center, The Affiliated Changzhou Second People’s Hospital of Nanjing Medical University, Changzhou, China

Correction to: *Scientific Reports* 10.1038/s41598-024-62116-w, published online 16 May 2024

The original version of this Article contained an error in Affiliation 5, which was incorrectly given as ‘Bone Disease Research and Clinical Rehabilitation Center, Changzhou Medical Center, Nanjing Medical University, Changzhou, China’. The correct affiliation is listed below:

Bone Disease Research and Clinical Rehabilitation Center, Changzhou Medical Center, The Affiliated Changzhou Second People’s Hospital of Nanjing Medical University, Changzhou, China.

The original Article has been corrected.

