# Supplementary material for: PPARγ regulates osteoarthritis chondrocytes apoptosis through caspase-3 dependent mitochondrial pathway
Source: Sci Rep. 2024 May 16;14:11237. doi: 10.1038/s41598-024-62116-w (PMC11099036; doi:10.1038/s41598-024-62116-w)
Supplement: Supplementary file 1 — Supplementary Information. [file 41598_2024_62116_MOESM1_ESM.docx]

Original blots

From cartilage tissues (The red boxes represent the blots used in the manuscript)


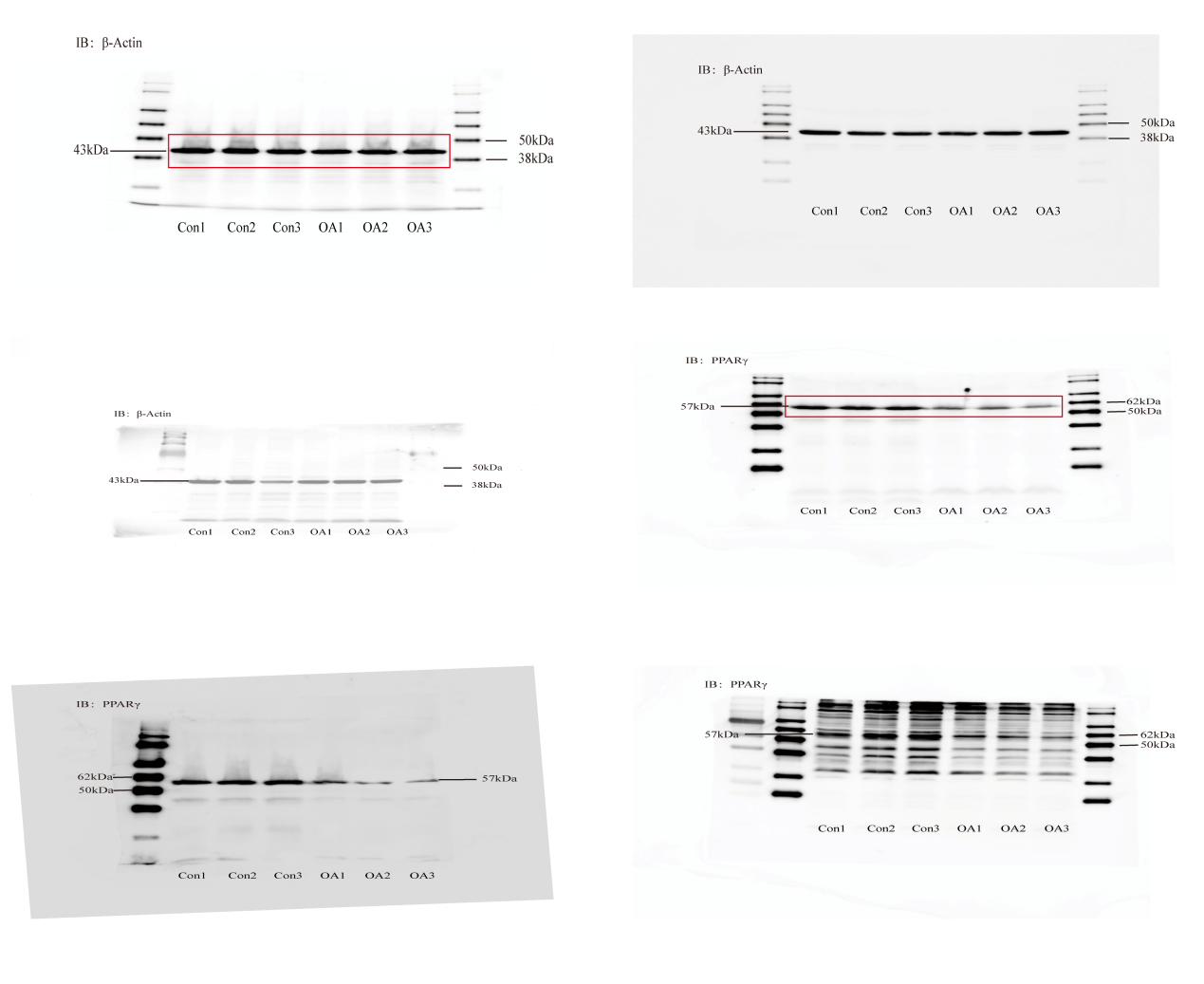


From chondrocytes (The red boxes represent the blots used in the manuscript)


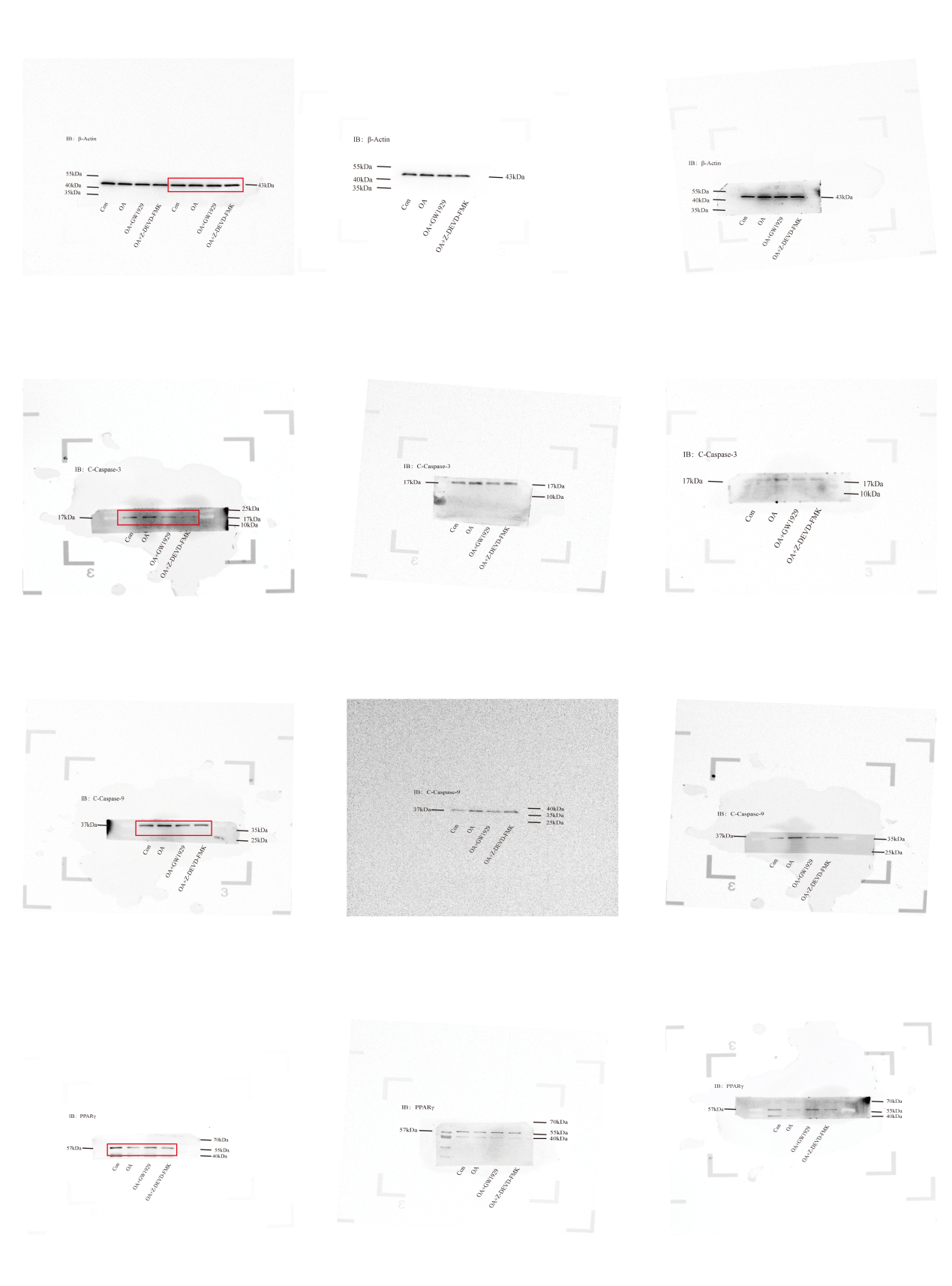


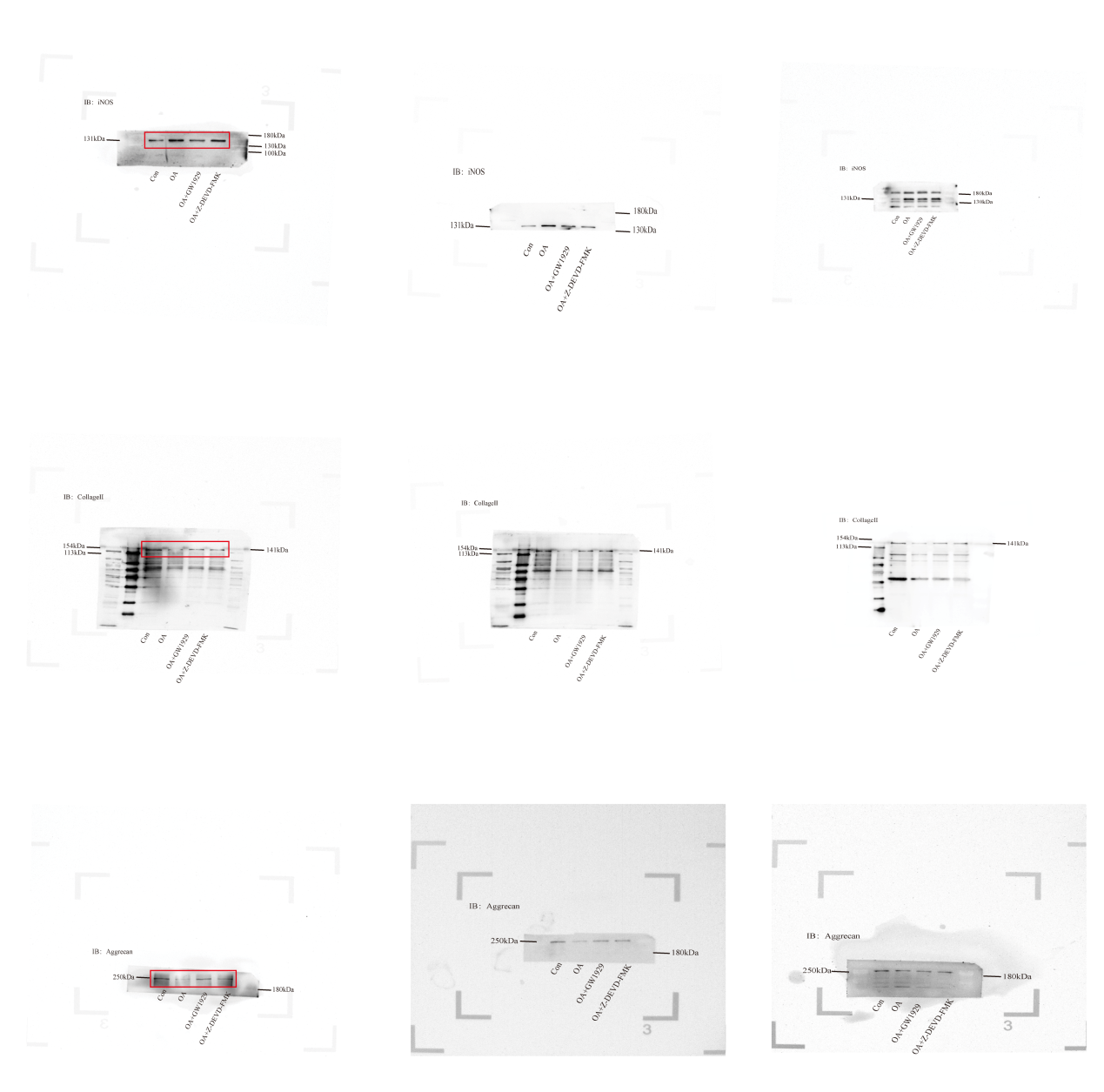


**Chromatin immunoprecipitation (ChIP)**

Cultured human normal chondrocytes (iCell Bioscience, Inc. Shanghai, China) were subjected to 10ng/ml IL-1β or pretreated with10μM GW1929 then 10ng/ml IL-1β or vehicle (PBS solution) for 12h. A total of 10^7^ cells were fixed and cross-linked in fresh 1% formaldehyde for 10min and then quenched with 2.5M glycine for 5min at room temperature. Cells were then harvested and suspended in lysis buffer. Simple ChIP Enzymatic Chromatin IP Kit (Cell Signaling Technology, Danvers, MA, USA) was used according to the manufacturer’s instructions. Chromatin was digested with micrococcal nuclease, sheared by sonication and then lysates were clarified by centrifugation at 10,000 rpm for 10min at 4°C. The supernatant was incubated with ChIP-grade rabbit antihuman PPARγ polyclonal antibody (Cell Signaling Technology, Danvers, MA, USA) or normal immunoglobulin G (IgG) overnight at 4°C with rotation. After being pulled down with protein G agarose beads, the target proteinDNA complexes were sequentially washed several times and reverse cross-linked to elute DNA for the subsequent experiment. DNA was purified and real-time PCR was performed to evaluate the ChIP-enriched DNA. Primer sequences for detecting PPARγ-binding iNOS promoter region were 5′- TTTATGACTGTGACTGCCAGGG -3′ (forward) and 5′- TGAACTGCCACCTTGGACTT -3′ (reverse).


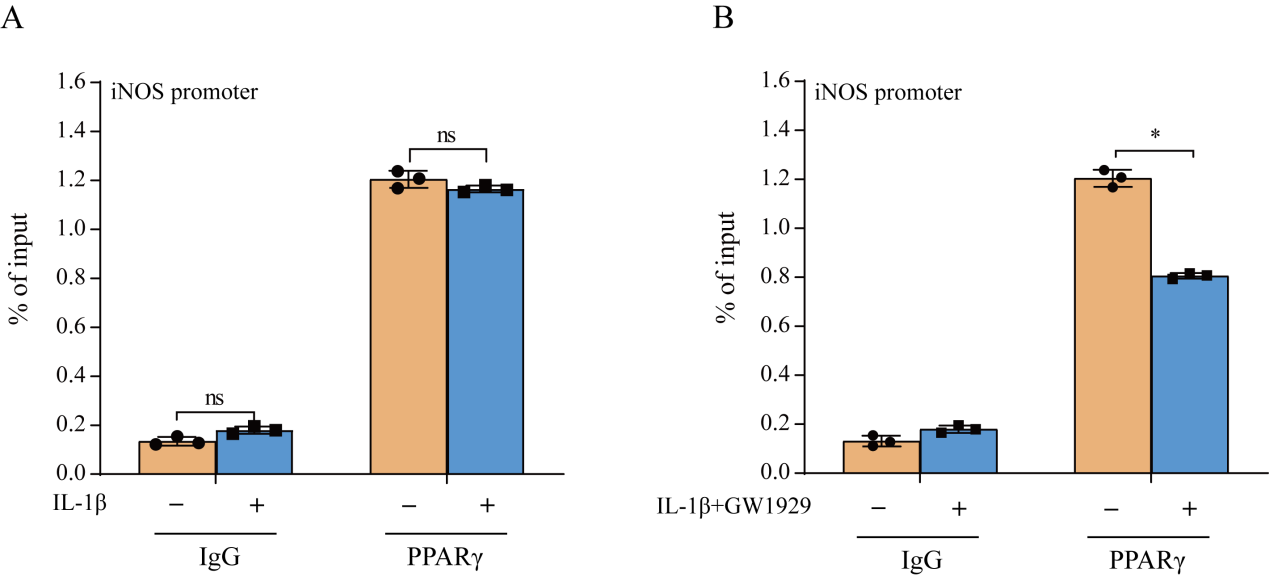


Figure S1. Human normal chondrocytes were treated for 12h, and then ChIP assay targeting the PPARγ binding site on iNOS was performed. Data were shown as the percentage of input. Results are presented as means±standard deviation of three independent experiments.

**P*<0.05.

**Terminal deoxynucleotidyl transferase dUTP nick-end labeling (TUNEL) staining**

TUNEL staining was performed using In Situ Cell Death Detection Kit according to the instruction of the manufacture. Briefly, the selected slices were dewaxed in xyleneand dehydrated in graded alcohol. After washing in 0.85%NaCl and PBS, the sections were fixed in the paraformaldehyde for 15min. 100μL of the 20ug/ml Proteinase K was added to each slide to cover the tissue section, which was incubated for 10min at room temperature. After washed in PBS, the tissue sections were fixed again in paraformaldehyde and 100μL equilibration buffer was added to each slide for 10min. 30μL of rTDT incubation buffer was added to each slide. The tailing reaction was performed in 37°C for 60min in a humidified chamber. After that, all performance was protected from light. Following sufficient wash, the sections were stained with DAPI. At last, sections were dehydration, clearing, and mounting with neutral gums. The sections were analyzed under the fluorescence microscope (CX41-32RFL, OLYMPUS, Tokyo, Japan).

.


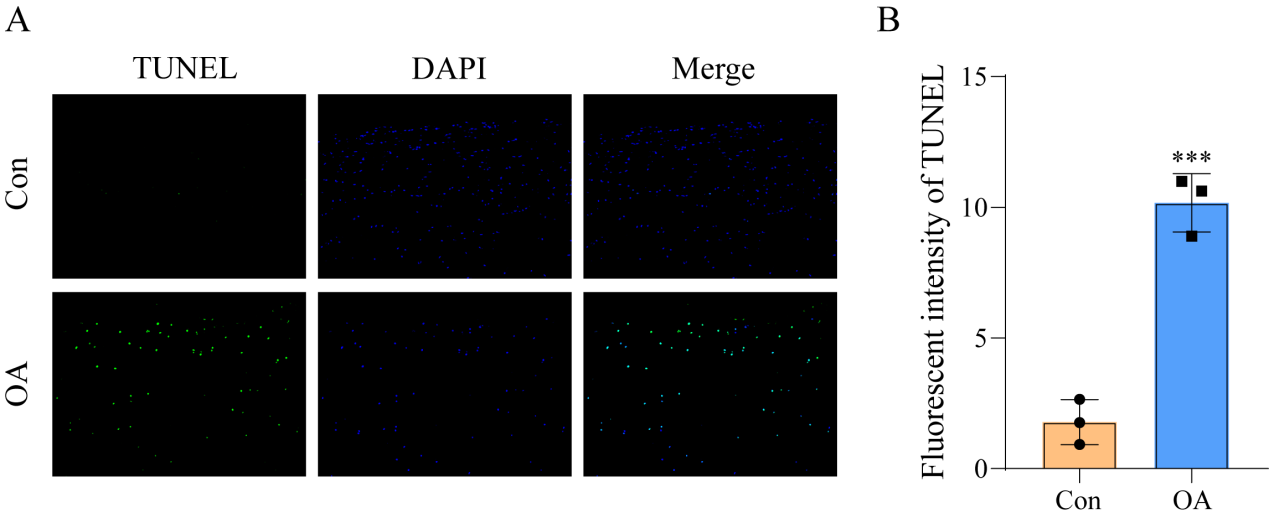


Figure S2. (A) Images of terminal deoxynucleotidyl transferase dUTP nick-end labeling (TUNEL) staining. (B) Fluorescence intensity. ****P*<0.001.

**Cell proliferation**

Cell proliferation was determined by 3-(4,5-dimethylthiazol-2-yl)-2, 5-diphenyltetra-zolium bromide reduction (MTT) assay (Beyotime Biotechnology, Shanghai, China). In brief, the indicated cells were seeded in 96-well plate at a density of 2×10^4^per well. Before the end of the experiment, 20μL MTT was added and the plate was incubated at 37°C for 4h. Subsequently, 150mL DMSO was added to dissolve formazan and the absorbance was measured at 570nm by the microplate reader (Elx808 Bio-Tek Instruments, Winooski, VT, USA).


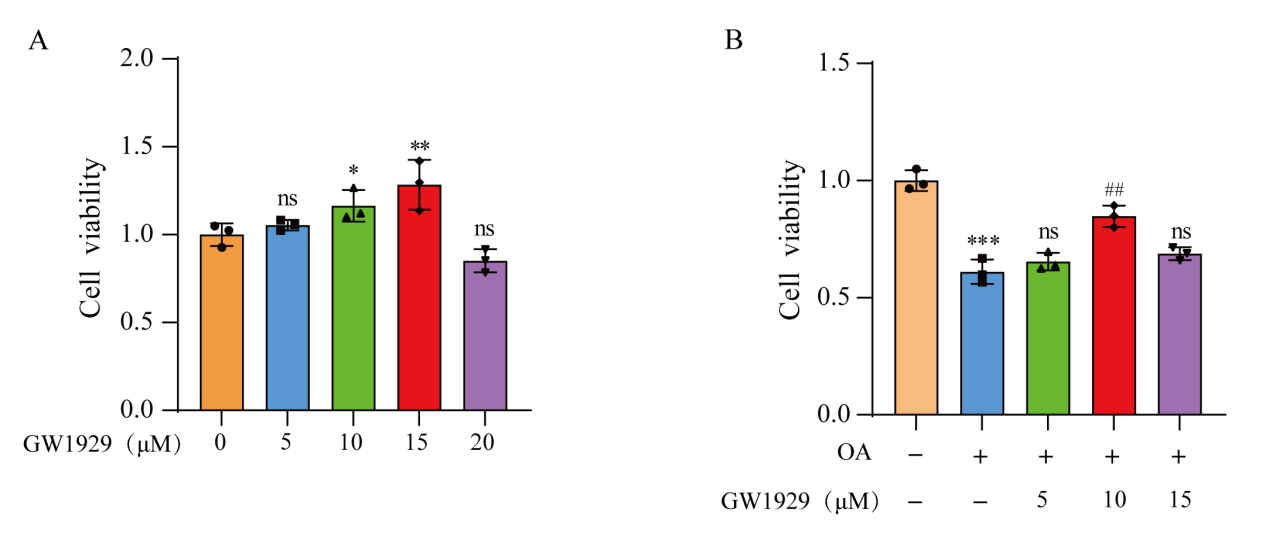


Figure S3. (A) Control chondrocytes were treated with different concentrations of GW1929 and cultured in vitro for 24h. (B) OA chondrocytes were treated with different concentrations of GW1929 and cultured in vitro for 24h. Results are presented as means±standard deviation of three independent experiments. **P*<0.05, ***P*<0.01, ****P*<0.001 versus Con. ^##^*P*<0.01 versus OA.


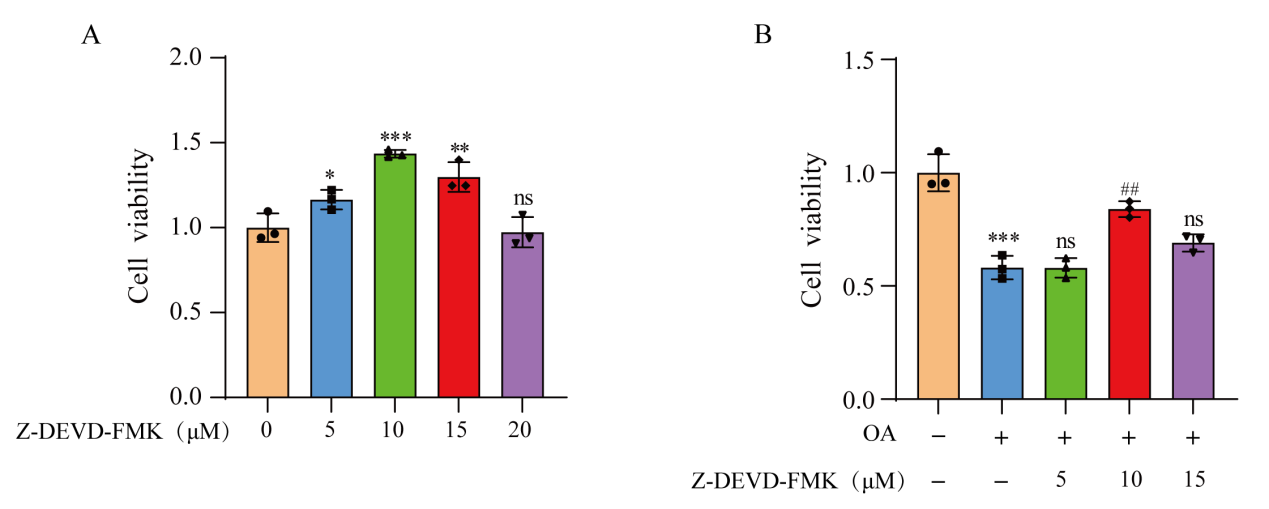


Figure S4. (A) Control chondrocytes were treated with different concentrations of Z-DEVD-FMK and cultured in vitro for 24h. (B) OA chondrocytes were treated with different concentrations of Z-DEVD-FMK and cultured in vitro for 24h. Results are presented as means±standard deviation of three independent experiments. **P*<0.05, ***P*<0.01, ****P*<0.001 versus Con. ^##^*P*<0.01 versus OA.
